# BoGDB: An integrative genomic database for *Brassica oleracea* L.

**DOI:** 10.3389/fpls.2022.852291

**Published:** 2022-08-24

**Authors:** Yong Wang, Jialei Ji, Zhiyuan Fang, Limei Yang, Mu Zhuang, Yangyong Zhang, Honghao Lv

**Affiliations:** Institute of Vegetables and Flowers, Chinese Academy of Agricultural Sciences, Beijing, China

**Keywords:** *Brassica oleracea* genome database, functional genomics, transcriptomics, metabolomics, *Brassica oleracea*

## Abstract

*Brassica oleracea* is an important species due to its high economic and nutritional value. Moreover, it is an ideal model for studies of morphology and genome evolution. In the genomic era, with massive “omics” data being generated, a high-efficiency platform is crucial to deepen our understanding of this important species. In this study, we developed the *B. oleracea* Genome Database (BoGDB) to consolidate genome, transcriptome, and metabolome data of *B. oleracea* cultivars, providing the first cross-omics platform for *B. oleracea*. In order to make full use of the multi-omics data, BoGDB integrates multiple functional modules, including “Gene Search,” “Heatmap,” “Genome Browser,” “Genome,” “Tools,” “Metabolic,” and “Variation,” which provides a user-friendly platform for genomic and genetic research and molecular design breeding of *B. oleracea* crops. In addition, BoGDB will continue to collect new genomic data of *B. oleracea* and integrate them into BoGDB when higher-quality genomic data are released.

## Introduction

*Brassica oleracea* is an economically essential cruciferous species, with about 100 million tons worldwide production in 2018.^1^ *B. oleracea* shows extreme morphological diversity, with various crop cultivars such as cabbage (*B. oleracea* var. *capitata*), broccoli (*B. oleracea* var. *italica*), cauliflower (*B. oleracea* var. *botrytis*), kale (*B. oleracea* var. *acephala*), Brussels sprouts (*B. oleracea* var. *gemmifera*), and kohlrabi (*B. oleracea* var. *gongylodes*), which are grown for their leaves, flowers, and stems.

*Brassica oleracea* cultivars have contributed to human health for hundreds of years and are popular for their high nutrition from carotenoids, dietary fibers and vitamins, and unique anticancer phytochemicals like indole-3-carbinol and sulforaphane. In addition, *B. oleracea* (CC genome, 2*n* = 18) is a unique model for evolution studies, as it experienced multiple polyploidy events and provides ancestor genomes of the two most important Brassica oil crops, *B. napus* (AACC) and *B. carinata* (BBCC).

Significant progress has been made in the field of *B. oleracea* genetics and genomics in the last decade. Liu et al. (2014) first published the draft genome of cabbage line 02-12, which has excellent agronomic traits. In the same year, Parkin et al. (2014) published the draft genome of TO1000, a doubled haploid kale-like variety. The assembly of these two genomes is done by next-generation genome assembly. Recently, third-generation sequencing technology has been used to complete the assembly and generate high-quality genomes of cabbage lines with different shapes (D134, JZS, and OX-heart), broccoli (HDEM), and cauliflower (Korso; Belser et al., 2018; Sun et al., 2019; Cai et al., 2020; Lv et al., 2020; Guo et al., 2021).

**Figure 1:**
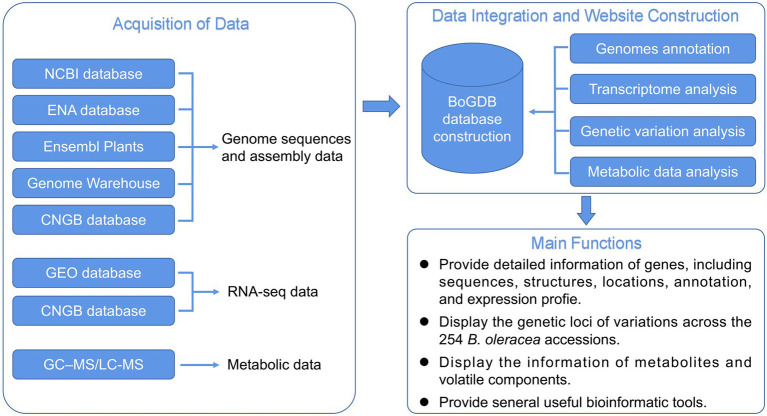
The flow diagram showing design and construction of BoGDB.

In addition to genome sequencing and assembly, research on transcriptomics, proteomics, and metabolomics in *B. oleracea* has revealed the gene expression, protein, and metabolite abundance profiles in various varieties (Liu et al., 2014; Parkin et al., 2014; Zhao et al., 2020; Wei et al., 2021). However, an integrated functional genomics database of multiple *B. oleracea* cultivars, enabling users to explore and use relevant omics data conjointly, is absent. Although the recently released BRAD V3.0 database (Chen et al., 2022) contains genomic data for many cruciferous species, there are only two species of *B. oleracea*, which could not satisfy our genomic analysis of *B. oleracea*. We thus designed the first integrative functional genomic database for *B. oleracea* (BoGDB),^2^ which integrates genome, transcriptome, and metabolome data of *B. oleracea*, providing a user-friendly platform for the study of *B. oleracea* (Figure 1).

## Construction of the BoGDB

### Acquisition of genomic, transcriptomic and metabolic data

Genome sequences of *B. oleracea* cultivar 02-12 were downloaded from DDBJ/EMBL/GenBank under the accession code AOIX00000000. Genome sequences of *B. oleracea* cultivar JZS (PRJCA001832) were downloaded from the Genome Warehouse database. Genome sequences of *B. oleracea* cultivars OX-heart 923 (PRJNA546441) and Korso (PRJNA548819) were downloaded from the National Center for Biotechnology Information (NCBI) database. Genome sequences of *B. oleracea* cultivar HDEM (PRJEB26621) were downloaded from the European Nucleotide Archive. Genome sequences of *B. oleracea* cultivars TO1000 were downloaded from the Ensembl Plants. Genome sequences of *B. oleracea* cultivars D134 (CNP0000469) were downloaded from the China National GeneBank (CNGB) database. Gene expression data (GSE42891) from different tissues of line 02-12, generated by next-generation sequencing, were acquired from the Gene Expression Omnibus (GEO) database. The full-length transcriptome data (CNP0001459) of five different organs of D134, obtained using Single-Molecule Real-Time (SMRT) sequencing, were downloaded from the CNGB database. The expression patterns of genes in response to Fusarium wilt and clubroot are displayed on BoGDB based on RNA-seq data (PRJNA548392, SRP144315) from the NCBI Sequence Read Archive. In addition, we collected cabbage metabolic data, including primary and secondary metabolites (Zhao et al., 2020) and volatile components (Wei et al., 2021), into BoGDB. The Omics data information in the BoGDB is shown in Table 1.

**Table 1:** Omics data information in the BoGDB.

**Data type**	**Cultivar**	**Description**
Genome	02-12	Genome sequences of round cabbage cultivar 02-12
Genome	D134	Genome sequences of round cabbage cultivar D134
Genome	JZS	Genome sequences of round cabbage cultivar JZS
Genome	OX-heart 923	Genome sequences of pointed cabbage cultivar OX-heart 923
Genome	Korso	Genome sequences of cauliflower cultivar Korso
Genome	HDEM	Genome sequences of broccoli cultivar HDEM
Genome	TO1000	Genome sequences of Chinese kale cultivar TO1000
Transcriptome	02-12	Gene expression data from seven different tissues of cabbage cultivar 02-12
Transcriptome	D134	Full-length transcriptome data of five different tissues of cabbage cultivar D134
Transcriptome	96–100, 01–20	RNA-seq data of cabbage resistant cultivar 96–100 and susceptible cultivar 01–20 after *Fusarium oxysporum* f. sp. *conglutinans* infection
Transcriptome	Xiangan336, Jingfeng No.1	RNA-seq data of cabbage resistant cultivar Xiangan336 and susceptible cultivar Jingfeng No. 1 after *Plasmodiophora brassicae* infection
Metabolome	DY2A, ZGF1	Metabolic data of primary and secondary metabolites
Metabolome	Guanjun, Jiuxing, Lvyu, Jindinghaoyue, Lixin285, Ziguang, Xinhonglu, Zijinyu, Tianzi17, Luyizihong265	Metabolic data of volatile components

### *Brassica oleracea* genomes annotation

InterProScan (Finn et al., 2017) was applied to localize large-scale protein function annotations of the gene-encoded protein sequences of seven *B. oleracea* genomes. The conserved domain feature data resources of the protein gene family included in Pfam (El-Gebali et al., 2019) and the hmmerscan command in the HMMER software (Finn et al., 2011) were used to identify the gene family to which the whole genome protein sequences belong. KEGG Mapper (Kanehisa et al., 2021) was used to annotate genes in batches to the KEGG pathway and obtained the visualized color pathway maps. The BlastKOALA (Kanehisa et al., 2016) annotation tool was used to analyze and obtain the corresponding KO annotation of the whole genome protein sequences. Use iTAK (Zheng et al., 2016) software to identify genome-wide transcription factors and protein kinases. All comment information is stored in tab-separated value TSV files.

### Transcriptome data analysis

Use the fastq-dump tool in the sratoolkit to further convert the original sequencing data into the standard fastq format. Fastp (Chen et al., 2018) was used for quality control filtering of data. Trimmomatic (Bolger et al., 2014) software was used to further filter the data that was still not ideal after fastp quality control filtering. After the quality control and filtering of the original sequencing data were completed, the sequencing data were compared to the corresponding genome using STAR (Sahraeian et al., 2011; Au et al., 2017). Then use RSEM software (Li and Dewey, 2011; Au et al., 2017) to construct the quantitative expression of all genes in the reference genome and stitch the corresponding expression matrix.

### Data integration and website construction

This research used Huawei Cloud Linux server as the basic environment for database development and deployment. After the above-mentioned data was standardized, it was stored in the relational database MySQL in the Linux cloud server (Figure 1). Then the database was built under the Linux development environment and the flask development framework based on the Python programming language. The front-end webpage development technology of the database is composed of HTML, CSS, and JavaScript language, supplemented by the Echarts package for data visualization, the Bootstrap front-end template development framework that can quickly write webpage modules, and the jQuery library that simplifies the JavaScript language. An online platform for BLAST sequence similarity retrieval was established using SequenceServer software (Priyam et al., 2019). Using JBrowse software (Buels et al., 2016) and Nginx reverse proxy server to integrate *B. oleracea* genome data, a high-performance genome browser was deployed to visually display genome sequences and corresponding annotation information. A high-performance FTP download station was deployed using vsftpd. Finally, in order to make the *B. oleracea* genomics information database accessible to the majority of researchers from the Internet, we used Gunicorn and Nginx to share the developed information database on the Internet.

## Utilization of the BoGDB

### The homepage of BoGDB

**Figure 2:**
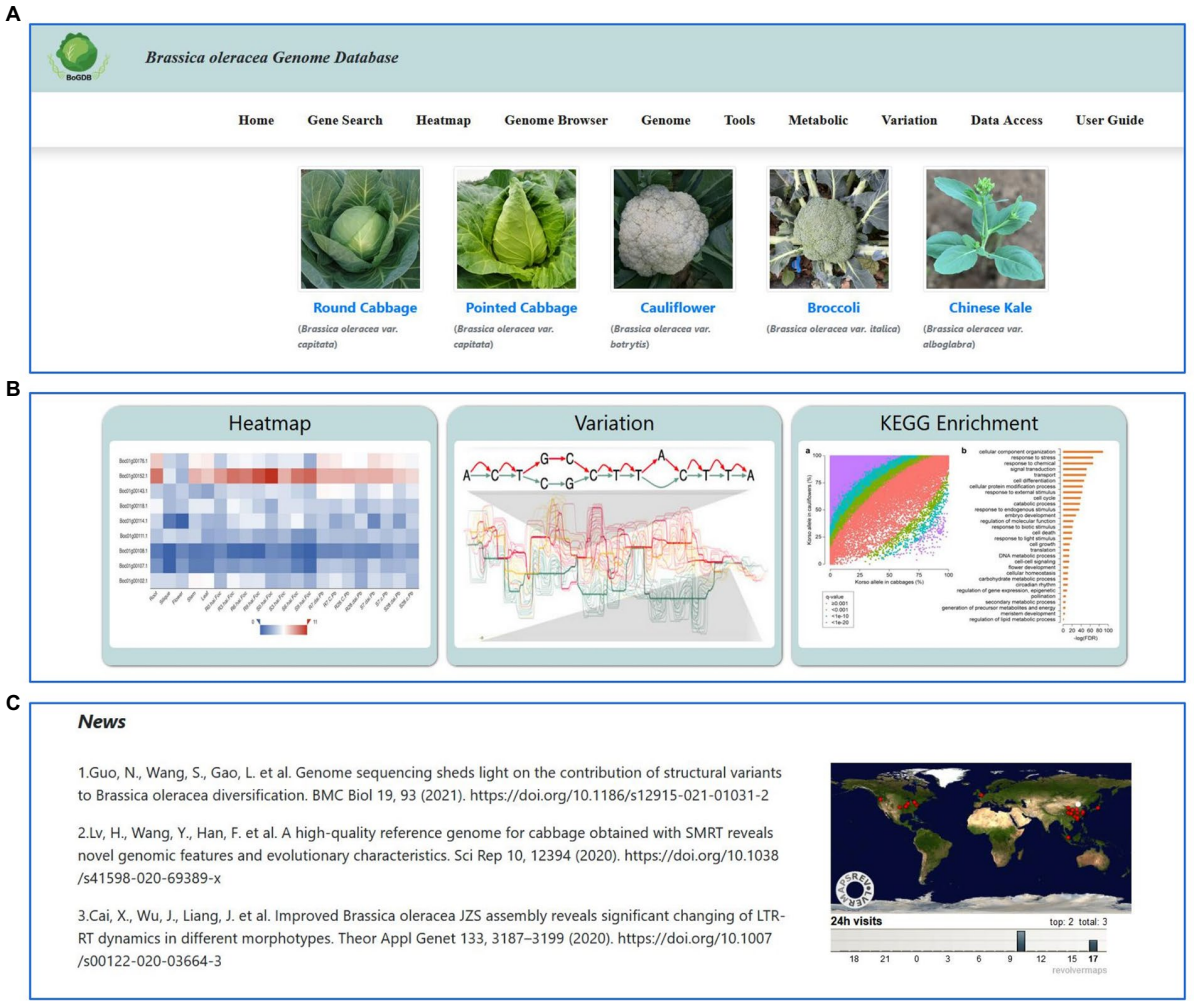
The homepage of BoGDB. **(A)** Navigation bar and species atlas. **(B)** Commonly used tool set. **(C)** News, citations, and global access.

**Figure 3:**
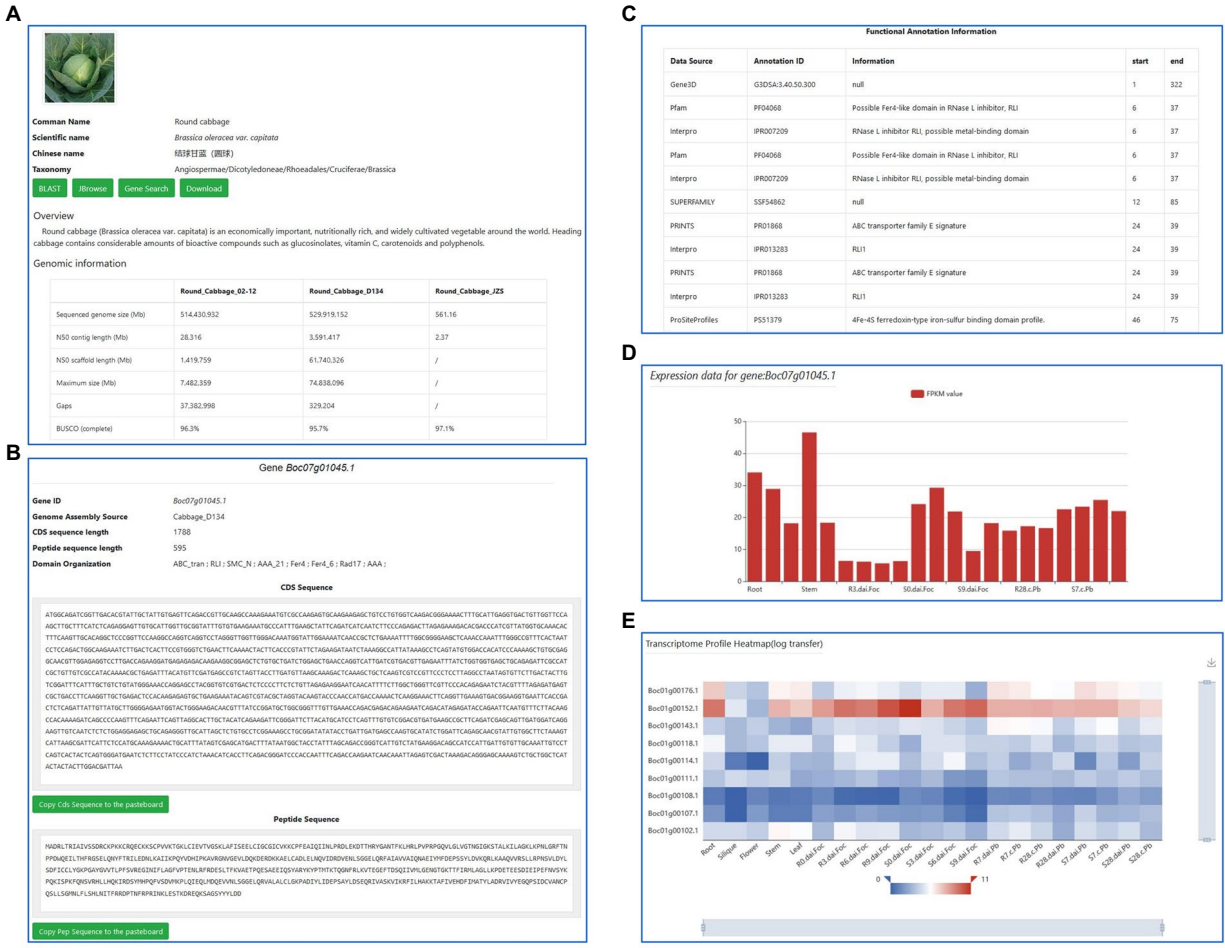
The “Gene search” and “Heatmap” modules. **(A)** The cultivar introduction and genome information. **(B–D)** The “Gene Search” module provides detailed gene information, including CDS, peptide sequence, annotation, and expression. **(E)** The dynamic, editable heatmap.

The homepage of BoGDB is mainly divided into 4 main parts: navigation bar, species atlas, commonly used tool set, and other columns (Figure 2). The navigation bar located at the top of the homepage consists of 9 labels: Home, Gene Search, Heatmap, Genome Browser, Genome, Tools, Metabolic, Variation, Data Access and User Guide (Figure 2A). Below the navigation bar is the cultivar atlas. Users can view the cultivar description and genome information by clicking on the name below the image (Figure 3A). Three commonly used toolkits, Heatmap, Variation and KEGG Enrichment, are given below the cultivar atlas (Figure 2B). At the bottom of the web page are news, citations, and global access (Figure 2C).

### The “Gene Search” and “Heatmap” modules

In the “gene search” module, users can view the detailed information of genes, including the coding sequence (CDS), peptide sequence, functional annotation information, and expression data by entering the ID of a gene of interest (GOI) in the ‘Gene ID Input’ area (Figures 3B–D). All sequences can be downloaded by choosing “Copy Cds/Pep Sequence to the clipboard.” The dynamic, editable heatmap generated from the differential expression analysis in cabbage cultivars 02-12 and D134 can be viewed when uploading the GOI list (Figure 3E). Moreover, it allows users to export the visualizations and the transcriptome profile matrix data.

### The “Genome Browser” and “Genome” modules

**Figure 4:**
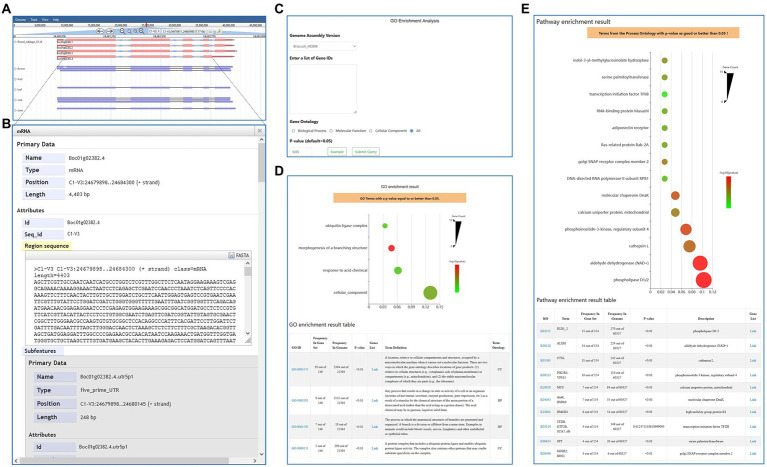
The “Genome Browser” module and GO/KEGG tools. **(A,B)** The “Genome Browser” module provides genomic data, including gene structure, gene location, and genome and transcript sequences. **(C,D)** The “GO Enrichment” shows enriched GO terms within a query gene list. **(E)** The “KEGG Enrichment” shows enriched KO terms within a query gene list.

The ‘Genome Browser’ module is an integrated tool for visualizing genomic data, which provides access to the gene structures, gene locations, as well as genomic and coding sequences (Figures 4A,B). Additionally, alternatively, spliced isoforms can be viewed based on an alignment with the full-length transcriptome data of D134. The ‘Genome’ module contains ‘BLAST’, ‘JBrowse’, ‘Gene Search’, and ‘Download’ and provides an overview of the seven cultivars and their reference genome assembly information.

### The “Tools” module

The “Tools” module is equipped with several popular bioinformatic tools for “BLAST,’” “Gene Family Search,” “Transcription Factor Search,” “Protein Kinase Search,” “Flanking Sequence Finder” “GO Enrichment,” and “KEGG Enrichment.” The “BLAST” tool supports pasting query DNA or protein sequences and dragging and dropping of fasta files; then, users can conduct a homology search in the preformatted genome database. “Gene Family Search,” “Transcription Factor Search,” and “Protein Kinase Search” are three search tools for searching gene family, transcription factor, and protein kinase by entering gene family name/PFAM ID, transcription factor name, and protein kinase name, respectively. Moreover, the ‘Flanking Sequence Finder’ is designed to assist users in finding the upstream and downstream sequence of GOIs, the length of which can be set up optionally. The ‘GO Enrichment’ and ‘KEGG Enrichment’ tools can identify the enriched or depleted Gene Ontology (GO) /KEGG Ontology (KO) terms within a query gene list and their corresponding *p*-values (Figures 4C–E).

### The “Metabolic” and “Variation” modules

**Figure 5:**
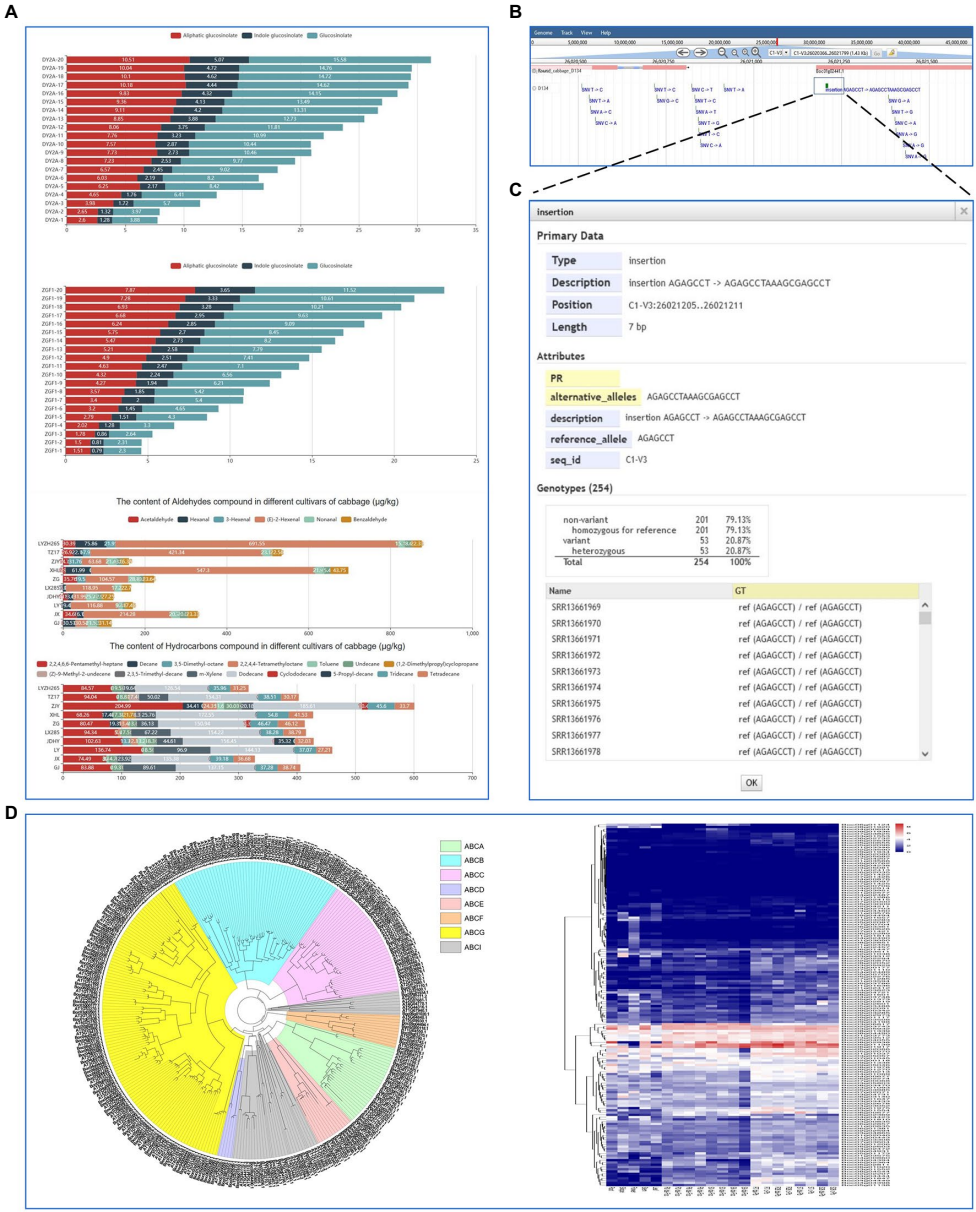
**“**Metabolic” and “Variation” modules and a case study for the application of BoGDB. **(A)** The “Metabolic” module presents the information of primary and secondary metabolites and volatile components. **(B)** The “Variation” module displays the genetic loci of SNPs and InDels across the 254 *B. oleracea* accessions. **(C)** Detailed information of variation sites. **(D)** A case study for the application of BoGDB.

The ‘metabolic’ module displays the information of primary and secondary metabolites and volatile components such as aldehydes, hydrocarbons, esters, alcohols, and ketones (Figure 5A). Users can easily obtain the genetic variations of desired genes with the ‘variation’ module. It displays the genetic loci of variations across the 254 *B. oleracea* accessions based on resequencing data from the NCBI database. The raw reads were aligned and mapped to the D134 reference genome using BWA and variants were called using BCFtools. Low-quality variants ‘QUAL <20 and DP < 5’ were removed using BCFtools filter. In addition, variants were annotated using snpEff. In total, we identified 2,818,621 single nucleotide polymorphisms (SNPs) and 396,413 insertions/deletions (InDels) using the D134 genome as a reference (Figures 5B,C).

### A case study for the application of BoGDB

Finally, we present an ATP-binding cassette (ABC) transporter gene family analysis using the BoGDB platform (Figure 5D). ABC transporters are a large and ancient family of transmembrane transport proteins that participate in the transport and accumulation of various substances, detoxification of harmful substances, stoma regulation, plant defense, and other physiological activities in the organism. A total of 162 ABC transporter genes were identified from the assembled genome of cabbage D134 by searching the PFAM ID ‘PF00005’ in the ‘Gene Family Search’ module. This result was consistent with the protein annotation information. A maximum-likelihood phylogenetic tree was constructed based on the ABC transporter protein sequences of *B. oleracea* and *Arabidopsis thaliana* using the FastTree program. The results show that the ABC transporter genes had been divided into eight subfamilies (A-H), with ABCG transporters constituting the largest subfamily. Moreover, we analyzed the expression differences of the ABC transporter genes of cabbage in different tissues in response to fusarium wilt and clubroot and created a heatmap. We found that some ABC transporter genes are differentially expressed related to disease resistance in cabbage. For instance, the expression of *Boc07g01045* and *Boc03g04460* was significantly upregulated in susceptible tissues after inoculation with *Fusarium oxysporum* f. sp. *Conglutinans,* and *Plasmodiophora brassicae*, respectively.

### Conclusion and future developments

*Brassica oleracea* is a unique species due to its high economic and nutritional value. Moreover, it is an ideal model for studies of morphology and genome evolution. In the genomic era, with massive “omics” data being generated, a high-efficiency and user-friendly platform is crucial to deepen our understanding of this important species. In this study, we developed BoGDB to consolidate genome, transcriptome, and metabolome data of *B. oleracea* cultivars, providing the first cross-omics platform for *B. oleracea*, which will significantly boost genomic and genetic research and molecular design breeding of these essential vegetable crops. In addition, BoGDB will continue to collect new genomic data of *B. oleracea* and integrate them into BoGDB when higher-quality genomic data are released.

## Data availability statement

The original contributions presented in the study are included in the article/supplementary material, further inquiries can be directed to the corresponding authors.

## Author contributions

HL, YZ, YW, and JJ conceived and designed the experiments. YW, JJ, ZF, LY, MZ, and YZ performed the experiments. YW, JJ, and HL wrote the manuscript. All authors contributed to the article and approved the submitted version.

## Funding

This work was supported by grants from the Central Public-interest Scientific Institution Basal Research Fund (Y2021XK18), the Science and Technology Innovation Program of the Chinese Academy of Agricultural Sciences (CAAS-ASTIP-IVFCAAS), and China Agriculture Research System of MOF and MARA.

## Conflict of interest

The authors declare that the research was conducted in the absence of any commercial or financial relationships that could be construed as a potential conflict of interest.

## Publisher’s note

All claims expressed in this article are solely those of the authors and do not necessarily represent those of their affiliated organizations, or those of the publisher, the editors and the reviewers. Any product that may be evaluated in this article, or claim that may be made by its manufacturer, is not guaranteed or endorsed by the publisher.

## Footnotes

^1^ http://faostat.fao.org/

^2^ http://www.bogdb.com
